# Adulterated Foil

**Published:** 1883-06

**Authors:** 


### ADULTERATED FOIL.

A bill is before the German Parliament to prohibit the use of any metallic foil which contains more than one per cent. of lead, in the packing and preserving of food of any kind offered for sale. It also prohibits the use of caoutchouc which contains lead or zinc, for the manufacture of nipples, baby-bottles, drinking cups, or playthings. Lead includes oxide and sulphate of lead; zinc includes the oxide of zinc. It is expected that the bill will be in operation after July 1.—*Zahntechnische Reform.*

